# Brief Report: Proatherogenic Cytokine Microenvironment in the Aortic Adventitia of Patients With Rheumatoid Arthritis

**DOI:** 10.1002/art.39574

**Published:** 2016-05-26

**Authors:** Ammad Ahmed, Ivana Hollan, Samuel A. Curran, Susan M. Kitson, Marcello P. Riggio, Knut Mikkelsen, Sven M. Almdahl, Pål Aukrust, Iain B. McInnes, Carl S. Goodyear

**Affiliations:** ^1^ University of GlasgowGlasgowUK; ^2^ Hospital for Rheumatic DiseasesLillehammerNorway; ^3^ University Hospital of North NorwayTromsøNorway; ^4^ Oslo University Hospital Rikshospitalet and University of OsloOsloNorway

## Abstract

**Objective:**

Patients with rheumatoid arthritis (RA) are at increased risk of developing cardiovascular disease (CVD) via mechanisms that have not yet been defined. Inflammatory pathways, in particular within the vascular adventitia, are implicated in the pathogenesis of primary CVD but could be amplified in RA at the local tissue level. The aim of this study was to examine the aortic adventitia of coronary artery disease (CAD) patients with or without RA to determine the cytokine profile contained therein.

**Methods:**

Aortic adventitia and internal thoracic artery biopsy specimens obtained from 19 RA patients and 20 non‐RA patients undergoing coronary artery bypass graft surgery were examined by immunohistochemistry.

**Results:**

Interleukin‐18 (IL‐18), IL‐33, and tumor necrosis factor (TNF) were expressed in aortic adventitia biopsy specimens from both groups, and expression of these cytokines was significantly higher in RA patients. In RA patients, IL‐33 expression in endothelial cells correlated positively with the number of swollen joints, suggesting a link between the systemic disease state and the local vascular tissue microlesion.

**Conclusion:**

The presence of the proinflammatory cytokines IL‐18, IL‐33, and TNF may play a role in the inflammatory process within the adventitia that contributes to plaque formation and destabilization. In theory, the amplified expression of these cytokines may contribute to the known increased occurrence and severity of CAD in patients with RA.

Rheumatoid arthritis (RA) is associated with an increased risk of cardiovascular disease (CVD), which cannot be completely explained by traditional risk factors 1. Cardiovascular events in patients with RA are characterized by greater severity in terms of recurrence and mortality. It is likely that both increased plaque formation and plaque vulnerability play roles in the increased CVD morbidity in patients with RA.

The proposed mechanisms of initiation and perpetuation of RA as well as atherosclerosis are multifactorial and have yet to be fully elucidated. Both diseases share immunologic/inflammatory components in their postulated pathology. Inflammation plays an important role in all stages of the atherosclerotic process (from endothelial cell [EC] activation to plaque formation, destabilization, and thrombosis) 2. Levels of systemic inflammatory biomarkers, including C‐reactive protein (CRP), tumor necrosis factor (TNF), and interleukin‐18 (IL‐18), predict CVD and/or disease progression in the general population. Consequently, there has been increasing interest in the role of cytokines in vascular disease, and the systemic levels of many cytokines, including IL‐33, have been investigated in this context. It should be recognized, however, that the role of circulating IL‐33 is complicated and thought to be situation dependent.

In mouse models of RA, administration of exogenous IL‐33 enhances disease activity, while in models of atherosclerosis it is protective 3, 4, 5. Moreover, the receptor for IL‐33, soluble ST2, is emerging as a novel biomarker of adverse vascular outcome. Administration of excess soluble ST2 has been shown to exacerbate atherosclerosis in mice 3, suggesting that expression of circulating IL‐33 in the local lesion may indeed be protective.

We previously reported that among patients with CVD, those with inflammatory rheumatic diseases have a higher frequency of inflammatory infiltrates in their aortic adventitia 6. Furthermore, compared with the occurrence of inflammatory infiltrates in the aorta, the occurrence of the adventitial infiltrates was significantly lower in the internal thoracic artery, which is a vessel that is highly resistant to atherosclerosis. Adventitial inflammation involving the vasa vasorum may play a substantial role in plaque formation, progression, and destabilization and contribute to the increased CV risk in RA. Consistent with this notion, current research has indicated a reduction in CV morbidity or mortality in patients who are treated effectively with antirheumatic therapies, which might be at least partially mediated through inhibition of vascular inflammation. In support of this concept, a recent study demonstrated significant attenuation of vascular inflammation in RA patients who received anti‐TNF therapy 7. Thus, with the introduction of new immunologic therapies, it is essential to examine which immune factors are involved in the vascular inflammatory process, because this may have implications for prevention and treatment of CVD.

We hypothesized that there is an amplified cytokine milieu within vascular lesions in patients with RA compared with controls, thus providing a potential tissue‐based explanation for the accelerated vascular risk in RA. Therefore, the aim of this study was to examine inflammatory cytokines that have plausible bioeffects in the vasculature in the aortic adventitia of CVD patients with or without RA and to evaluate their relationship to informative clinical parameters. We elected to focus on IL‐18, IL‐33, and TNF, because these cytokines have been implicated in the pathology of both atherosclerosis and RA.

## PATIENTS AND METHODS

### Patients

Among patients undergoing coronary artery bypass graft (CABG) surgery in the Feiring Biopsy Heart Study 6, we selected 19 patients who fulfilled the American College of Rheumatology 1987 revised criteria for the classification of RA 8 and 20 random control subjects. All of the patients gave written informed consent. The regional Ethics Committee for Medical Research Norway approved the study. The demographic and clinical characteristics of the patients are shown in Table 1.

**Table 1:** Characteristics of the patients^a^

Characteristic	RA (n = 19)	Non‐RA (n = 20)	*P*
Age, years	69 ± 9.1	68 ± 9.5	0.70
Male, no. (%)	12 (63)	14 (70)	0.74
Duration of CAD, years	6.3 ± 8.7	7.2 ± 6.6	0.39
History of myocardial infarction, no. (%)	15 (79)	9 (45)	0.05
Acute coronary syndrome, no. (%)	9 (47)	4 (20)	0.10
Time from angiography to CABG, days	17 ± 30	31 ± 60	0.99
Left ventricular ejection fraction	59 ± 13	64 ± 13	0.16
Hypertension, no. (%)	13 (68)	9 (45)	0.20
Family history of CAD, no. (%)	11 (58)	20 (100)	0.001
Hyperlipidemia, no. (%)	16 (84)	20 (100)	0.11
Duration of RA, years	19 ± 15	–	–
Patient's global assessment of IRD (0–100‐mm VAS)	29 ± 24	–	–
No. of swollen joints (28 assessed)	2.2 ± 2.6	0	0.002
Disease Activity Score in 28 joints	3.2 ± 1.0	–	–
Rheumatoid factor, IU/ml	316 ± 568	–	–
CCP, units/ml	553 ± 667	–	–
C‐reactive protein, mg/liter	20 ± 39	3.1 ± 3.1	0.02
ESR, mm/hour	34 ± 29	15 ± 9.5	0.06
Body mass index, kg/m^2^	25 ± 5.0	25 ± 2.4	0.78
Diabetes, no. (%)	2 (11)	1 (5)	0.61
Previous smoker, no. (%)	10 (52)	12 (60)	0.75
Current smoker, no. (%)	2 (11)	1 (5)	0.61
Current treatment, no. (%)			
Oral corticosteroids	8 (42)	0 (0)	0.001
Disease‐modifying drugs	16 (84)	0 (0)	<0.0001
COX‐2–selective inhibitors	5 (26)	0 (0)	0.02
Traditional NSAIDs	2 (11)	0 (0)	0.23
Lipid‐lowering drugs	16 (84)	19 (95)	0.34
Acetylsalicylic acid	17 (90)	17 (85)	1.0
Beta‐blockers	15 (88)	14 (74)	0.41
ACE inhibitors	5 (31)	5 (25)	0.47

a Fisher's unconditional exact test was used to assess differences in proportions between the 2 groups. Differences in continuous variables between the 2 groups were assessed by *t*‐test for independent samples (age, body mass index), Mann‐Whitney U test (duration of coronary artery disease [CAD], time from angiography to coronary artery bypass graft [CABG], left ventricular ejection fraction, erythrocyte sedimentation rate [ESR]), and Wilcoxon's signed rank test (no. of swollen joints). Except where indicated otherwise, values are the mean ± SD. RA = rheumatoid arthritis; IRD = inflammatory rheumatic disease; VAS = visual analog scale; CCP = cyclic citrullinated peptide; COX‐2 = cyclooxygenase 2; NSAIDs = nonsteroidal antiinflammatory drugs; ACE = angiotensin‐converting enzyme.

### Biopsy and immunohistochemistry

We examined specimens from the aortic adventitia (including the aortic portion of the epicardial layer) and the internal thoracic artery, which were removed during CABG surgery. A section from the aortic adventitia (∼5 × 10 mm) covered by the periaortic portion of the epicardium was removed from the ventral aspect of the ascending aorta, in connection with the establishment of the proximal aortocoronary anastomoses 6. For the safety of the patients, these anastomoses are made in (and, therefore, the biopsy specimens are obtained from) areas with less pronounced atherosclerosis. A section of tissue from the internal thoracic artery (5–10 mm long) was obtained from the distal portion of the internal thoracic artery, while the proximal portion was used as a graft 9. The biopsy specimens were fixed in formalin, embedded in paraffin, and cut in 3‐μm–thick sections. Some findings regarding the aortic adventitia and internal thoracic artery used in this study have been reported previously 6, 9.

### Immunohistochemical analysis

Slides were prepared as previously described 6. Briefly, depending on the antibody (additional information is available upon request from the corresponding author), slides were preblocked with 0.5% hydrogen peroxide followed by antigen retrieval (using the heat‐induced citrate antigen retrieval or trypsin antigen retrieval protocol). After blocking with 5% normal horse serum with or without biotin, tissue sections were incubated with primary antibodies (CD68, IL‐18, IL‐33, and TNF) overnight, followed by 0.5% hydrogen peroxide if required. Horseradish peroxidase***–***conjugated or biotin‐conjugated secondary antibodies were incubated for 1 hour, followed by avidin***–***biotin‐peroxidase complex macromolecular complex (Vector). Binding was visualized with ImmPACT DAB Peroxidase Substrate (Vector). Slides were counterstained with hematoxylin.

### Scoring of stained tissues

Slides were semiquantitatively scored in random order by researchers who were blinded with regard to disease. CD68 staining was scored as follows: 0 = no cells, 1 = <20 cells, 2 = 21**–**50 cells, 3 = 51**–**99 cells, 4 = 100**–**199 cells, and 5 = >200 cells per section. IL‐18 and TNF staining was scored as follows: 0 = 0% of section, 1 = <10% of section, 2 = 10**–**25% of section, and 3 = >25% of section. IL‐33 staining was scored both according to the percentage of IL‐33**–**positive vasa vasorum per section and according to the number of IL‐33**–**positive ECs per vasa vasorum.

### Soluble autoantibody and cytokine receptor detection

All serum samples were collected prior to CABG, after a minimum of 4 hours of fasting. Enzyme‐linked immunosorbent assay, using appropriately diluted serum according to the instructions of the manufacturer, was performed to assay titers of soluble ST2 (R&D Systems) and anti–cyclic citrullinated peptide 2 (anti–CCP‐2; Axis‐Shield). A BN Systems N Latex RF kit (Siemens) was used to determine rheumatoid factor (RF) levels in serum samples.

### Statistical analysis

GraphPad Prism and SPSS software were used for all statistical analyses. The chi‐square test and Fisher's exact test (for categorical variables), the *t*‐test for independent samples (for continuous normally distributed variables), and the Mann‐Whitney U test (for continuous variables without normal distribution) were used to identify significant differences between RA and non‐RA patients. Correlations were determined using Spearman's rho. *P* values less than 0.05 were considered significant, and all tests were 2‐sided.

## RESULTS

### Macrophage detection and IL‐18 and TNF expression in aortic adventitia

**Figure 1:**
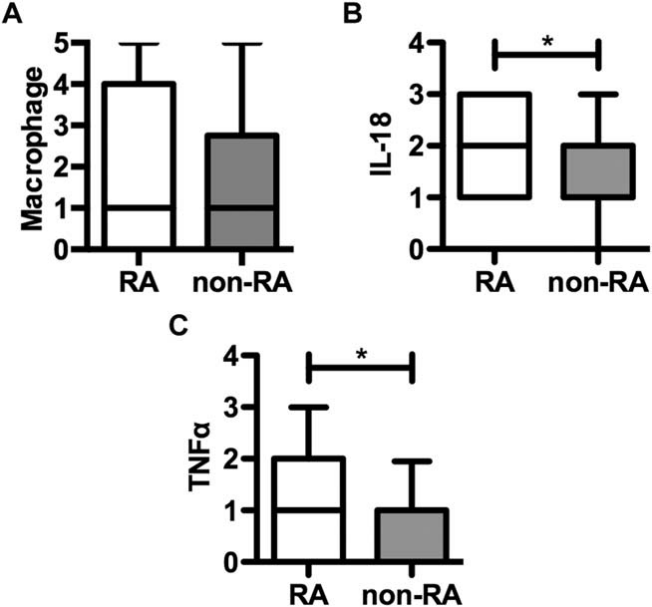
Macrophage, interleukin‐18 (IL‐18), and tumor necrosis factor (TNF) evaluation in the aortic adventitia of late‐stage cardiovascular disease (CVD) patients with (n = 19) and those without (n = 20) rheumatoid arthritis (RA). CD68+ macrophages (**A**), IL‐18 (**B**), and TNF (**C**) were evaluated immunohistologically, and the results were scored as described in Patients and Methods. Data are shown as box plots. Each box represents the 25th to 75th percentiles. Lines inside the boxes represent the median. Lines outside the boxes represent the 5th and 95th percentiles. ∗ = *P* < 0.05.

The proportion of aortic adventitia biopsy specimens in which CD68+ macrophages were detectable (63% of RA specimens and 56% of non‐RA specimens) and the number of CD68+ cells per section (Figure 1A) were similar in CVD patients with RA and those without RA (additional information is available upon request from the corresponding author). TNF was expressed in 63% of biopsy specimens from RA patients compared with 30% of specimens from non‐RA patients (*P* = 0.04). In contrast, IL‐18 was present in all biopsy specimens, suggesting ubiquitous expression in the aortic vascular tissue lesion (additional information is available upon request from the corresponding author). Crucially, although no difference in the number of macrophages between RA and non‐RA patients was observed (Figure 1A), the absolute level of IL‐18 and TNF expression in aortic adventitia was higher in RA patients (Figures 1B and C) compared with non‐RA controls (*P* = 0.03 and *P* = 0.02, respectively).

Neither the number of macrophages nor the level of IL‐18 or TNF expression correlated with hypertension or smoking (additional information is available upon request from the corresponding author). Furthermore, the expression of IL‐8 and TNF did not correlate with several markers of CVD severity, including the number of coronary arteries with significant stenosis, New York Heart Association (NYHA) class 10, or the number of previous myocardial infarctions. However, in RA patients, the left ventricular ejection fraction (as assessed by ventriculography) was negatively correlated with IL‐18 levels (r = −0.569, *P* = 0.03) and the macrophage number (r = −0.587, *P* = 0.05). In RA patients, neither the macrophage number nor cytokine expression correlated with several markers of disease severity, disease activity (i.e., the Disease Activity Score in 28 joints [11], tender joint count, erythrocyte sedimentation rate, CRP, RF, or anti–CCP‐2 level), or disease duration.

### IL‐33 in ECs within aortic adventitia

**Figure 2:**
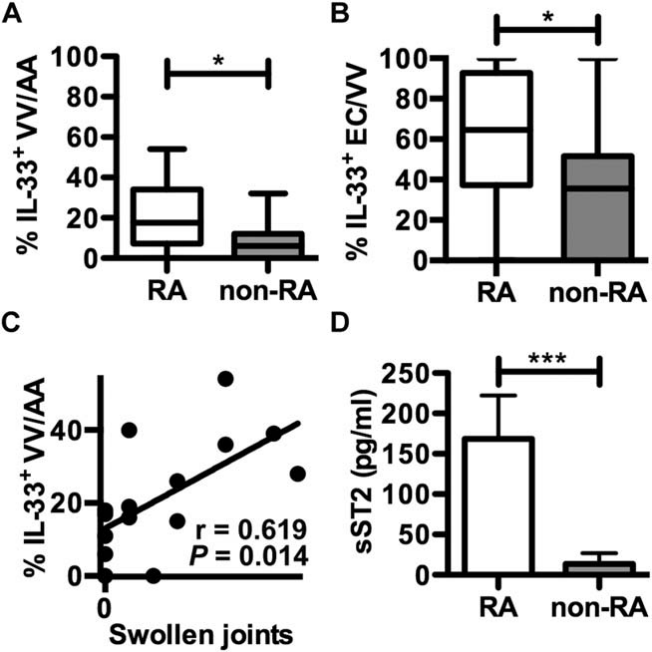
IL‐33 and soluble ST2 (sST2) in late‐stage CVD patients with (n = 19) and those without (n = 20) RA. **A** and **B,** Immunohistologic evaluation and quantification of the percentage of vasa vasorum (VV) in the aortic adventitia (AA) that were positive for IL‐33 (**A**) and the percentage of IL‐33–positive endothelial cells (ECs) in each vasa vasorum (**B**). Data are shown as box plots. Each box represents the 25th to 75th percentiles. Lines inside the boxes represent the median. Lines outside the boxes represent the 5th and 95th percentiles. **C,** Correlation between swollen joint counts and the percentage of IL‐33–positive ECs in each vasa vasorum. **D,** Serum levels of soluble ST2. Values are the mean ± SEM. ∗ = *P* < 0.05; ∗∗∗ = *P* = 0.002. See Figure 1 for other definitions.

IL‐33 was detected in the majority of aortic adventitia biopsy specimens from both RA patients (88%) and non‐RA patients (72%) but was restricted to the nucleus of vasa vasorum ECs (additional information is available upon request from the corresponding author). The proportion of vasa vasora expressing IL‐33 (IL‐33+ vasa vasorum) was higher in RA patients compared with non‐RA patients (mean ± SEM 20.4 ± 15.8% versus 8.6 ± 9.5%; *P* = 0.02) (Figure 2A). The proportion of EC nuclei positive for IL‐33 within each vasa vasorum (IL‐33+ ECs) was also substantially greater in RA patients compared with non‐RA patients (mean ± SEM 58.7 ± 34.1% versus 32.9 ± 28.5%; *P* = 0.02) (Figure 2B). In the internal thoracic artery, the proportion of IL‐33+ vasa vasorum was similar in RA patients (89%) and non‐RA patients (79%), but RA patients had a higher proportion of IL‐33+ ECs (30.6 ± 26.3% versus 13.1 ± 12.5%; *P* = 0.04). The proportions of IL‐33+ ECs and IL‐33+ vasa vasorum in aortic adventitia did not correlate with any traditional CV risk factors (additional information is available upon request from the corresponding author). In RA patients, the proportion of IL‐33+ ECs in aortic adventitia was significantly correlated with NYHA functional class (r = 0.618, *P* = 0.018) but not with other markers of CVD severity. Furthermore, the proportion of IL‐33+ ECs in the aortic adventitia of RA patients was also significantly correlated with the tender joint count (r = 0.582, *P* = 0.023) (data not shown) and the swollen joint count (r = 0.619, *P* = 0.014) (Figure 2C).

Interpretation of the functional implications of circulating IL‐33 expression generally requires parallel evaluation of soluble ST2. Even though we observed nuclear IL‐33 expression only in ECs and not extracellularly, we evaluated soluble ST2 levels in serum and observed significantly higher levels in RA patients compared with non‐RA patients (mean ± SEM 158.5 ± 52 pg/ml versus 12.79 ± 13 pg/ml; *P =* 0.002) (Figure 2D). The level of soluble ST2 did not correlate with vascular IL‐33 expression or other RA clinical and immunologic parameters; however, it did correlate with IL‐18 expression (r = 0.582, *P* = 0.02) and the CRP level (r = 0.745, *P* = 0.001).

## DISCUSSION

Our novel study demonstrates a significant alteration in the aortic adventitia cytokine microenvironment in CAD patients with RA compared with those without RA. Our data suggest that the underlying pathology in the vessel is different not only at a local level (i.e., ECs of microvessels within aortic adventitia) but also systemically within other macrovessels (i.e., internal thoracic artery). We observed that compared with non‐RA controls, RA patients have significantly higher expression of the proinflammatory cytokines IL‐18 and TNF in aortic adventitia, higher expression of nuclear IL‐33 in ECs of vasa vasorum in aortic adventitia, and higher levels of soluble ST2.

Although most studies have focused on the expression of circulating IL‐33, previous studies showed that IL‐33 was expressed in human vascular ECs 3, 12, and this may be of great importance. Intracellular/nuclear full‐length IL‐33, which is thought to act as an alarmin when it is released by necrotic cells 13, is a transcription factor 14. As a transcription factor, endothelial IL‐33 can induce NF‐κB signaling, with subsequent EC activation (i.e., up‐regulation of vascular cell adhesion molecule [VCAM] and intercellular adhesion molecule [ICAM]) 15. Thus, the vasa vasora of RA patients with increased nuclear IL‐33 are likely to be more predisposed to EC activation, leading to increased adhesion and infiltration of inflammatory cells, which again will increase IL‐33 expression.

This is further supported by the fact that TNF (which was increased in aortic adventitia in our RA cohort) is known to stimulate ECs, with a consequent up‐regulation of ICAM and VCAM via an intracellular IL‐33–dependent signal 15. The increased IL‐33 expression within microvascular ECs of RA patients might therefore represent a stromal alteration, which could contribute to increased CVD risk in RA. Furthermore, it is known that TNF, similar to other proinflammatory cytokines, increases secretion of soluble ST2 by numerous cell types 16. Thus, although soluble ST2 may counteract circulating IL‐33 activity, the high systemic levels of soluble ST2 in RA could reflect enhanced inflammation, which is supported by the observed correlation with the CRP level. Moreover, the correlation between soluble ST2 and aortic adventitia IL‐18 expression is also pertinent given that increased IL‐18 expression exacerbates atherosclerosis 17, while IL‐18 deficiency reduces atherosclerosis 18.

Collectively, our data suggest that in RA patients, the adventitial microenvironment, characterized by higher levels of IL‐18 and TNF and higher expression of nuclear IL‐33 within microvascular ECs, together with increased levels of soluble ST2, amplify the proatherogenic nature of the vessel, which theoretically enhances the pathology of atherosclerosis.

Based on the observed increased number of IL‐33+ ECs within both the aortic adventitia and the internal thoracic artery of RA patients, one might speculate that this phenomenon could lead to a generalized endothelial dysfunction and impairment of microvascular function, which is likely to contribute to both RA‐specific manifestations (such as synovitis and extraarticular manifestations) and the pathogenesis of ischemia (due to both macrovascular and microvascular disease) and heart failure. In theory, the observed positive association between IL‐33+ ECs and NYHA class may reflect, among other factors, increased ischemia in patients in whom the expression of IL‐33 in ECs is increased, causing microvascular cardiac disease (which is known to frequently occur in RA). It should also be noted that although the luminal endothelium has been the main focus of studies and theories to explain aspects of the pathogenesis of atherosclerosis, our studies suggest that the endothelium covering vasa vasorum (which provides the oxygen and nutrition supply to the macrovessel) may also play a crucial role in the development of atherosclerosis.

Of note, the number of macrophages and the level of IL‐18 expression in the aortic adventitia were inversely correlated with the left ventricular ejection fraction. The cause of this finding is unclear, and although it could be the result of a Type II error, it could reveal an important clinical relationship. For example, aortic inflammation might reflect systemic inflammation, which might contribute to the development of heart failure. Moreover, it is possible that aortic inflammation mirrors a more generalized vascular inflammation, including the coronary arteries (and small intramural cardiac arteries), which leads to myocardial ischemia and consequent ischemia‐related heart failure.

The current study has several possible limitations, which include a relatively small sample size. Hence, the lack of some associations and differences may be caused by Type II errors. However, compared with other biopsy studies, this study is relatively large and does provide justification for larger, more in‐depth studies. This is also a cross‐sectional study, and therefore no cause‐and‐effect relationships can be identified. However, it should be noted that this study is the first to examine the aortic adventitia microenvironment in RA and non‐RA patients and thus provides valuable information about vascular biology. Moreover, an additional advantage is that the current study did not rely on autopsy samples, which are susceptible to deterioration postmortem, but instead difficult‐to‐obtain fresh surgical specimens were used.

In summary, our data suggest that not only systemic inflammation but also vascular inflammation may play a role in the pathogenesis of CVD and of accelerated CVD in RA, with IL‐33 and related cytokines being potentially important players. These findings may help to explain why antirheumatic therapies, including anti‐TNF treatment, decrease the risk of CVD 7. In theory, these therapies may reduce the CV risk not only due to an effect on systemic inflammation but also due to a direct effect on the vasculature: both on inflammation in plaques, and on adventitial inflammation, which is suspected to be involved in both atheroma formation and destabilization. These results therefore emphasize the necessity for future studies that examine the influence of the vascular cytokine environment in RA on CVD outcome.

## AUTHOR CONTRIBUTIONS

All authors were involved in drafting the article or revising it critically for important intellectual content, and all authors approved the final version to be published. Dr. Goodyear had full access to all of the data in the study and takes responsibility for the integrity of the data and the accuracy of the data analysis.

### Study conception and design

Hollan, Riggio, Mikkelsen, Almdahl, Aukrust, McInnes, Goodyear.

### Acquisition of data

Ahmed, Hollan, Curran, Kitson, Goodyear.

### Analysis and interpretation of data

Ahmed, Hollan, Curran, McInnes, Goodyear.
